# Interaction of *Vibrio* spp. with the Inner Surface of the Digestive Tract of *Penaeus monodon*

**DOI:** 10.1371/journal.pone.0135783

**Published:** 2015-08-18

**Authors:** Wipasiri Soonthornchai, Sage Chaiyapechara, Padermsak Jarayabhand, Kenneth Söderhäll, Pikul Jiravanichpaisal

**Affiliations:** 1 Program in Biotechnology, Faculty of Science, Chulalongkorn University, Bangkok, 10330, Thailand; 2 Aquatic Molecular Genetics and Biotechnology Laboratory, National Center for Genetic Engineering and Biotechnology (BIOTEC), National Science and Technology Development Agency (NSTDA), 113 Paholyothin Rd., Klong 1, Klongluang, Pathumthani, 12120, Thailand; 3 Interdisciplinary Graduate Program on Maritime Administration, Graduate School, Chulalongkorn University, Bangkok, 10330, Thailand; 4 Department of Comparative Physiology, Uppsala University, Norbyvägen 18A, SE-752 36, Uppsala, Sweden; 5 Fish Vet Group Asia Limited, 99/386, Chaengwattana Rd., Toongsonghong, Laksi, Bangkok, 10210, Thailand; Johns Hopkins University, Bloomberg School of Public Health, UNITED STATES

## Abstract

Several species of *Vibrio* are the causative agent of gastroenteritis in humans. In aquaculture, *Vibrio harveyi* (*Vh*) and *V*. *parahaemolyticus* (*Vp*) have long been considered as shrimp pathogens in freshwater, brackish and marine environments. Here we show by using scanning electron microscopy (SEM) that *Penaeus monodon* orally inoculated with each of these two pathogens via an *Artemia* diet had numerous bacteria attached randomly across the stomach surface, in single and in large biofilm-like clusters 6 h post-infection. A subsequent marked proliferation in the number of *V*. *harveyi* within the biofilm-like formations resulted in the development of infections in the stomach, the upper and middle midgut, but neither in the posterior midgut nor the hindgut. SEM also revealed the induced production of peritrichous pili-like structures by the *Vp* attaching to the stomach lining, whilst only a single polar fibre was seen forming an apparent physical bridge between *Vh* and the host’s epithelium. In contrast to these observations, no such adherences or linkages were seen when trials were conducted with non-pathogenic *Vibrio* spp. or with *Micrococcus luteus*, with no obvious resultant changes to the host’s gut surface. In naive shrimp, the hindgut was found to be a favorable site for bacteria notably curved, short-rod shaped bacteria which probably belong to *Vibrio* spp. Data from the current study suggests that pathogens of *P*. *monodon* must be able to colonize the digestive tract, particularly the stomach, where chitin is present, and then they use an array of virulent factors and enzymes to infect their host resulting in disease. Oral infection is a better way of mimicking natural routes of infection; investigating the host-bacteria interactions occurring in the digestive tract may lead to new strategies for the prevention or control of bacterial infections in penaeids.

## Introduction

The human microbiome has been shown to increase the integrity of the intestinal mucosal barrier [1–3], to enable and educate the immune system [4, 5], to regulate the proliferation and differentiation of epithelial lineages [6, 7], to modulate angiogenesis [8], to modify the activity of the enteric nervous system to influence brain development and behaviour [9, 10], and, in helping the digestion of food, producing vitamins [11]. In aquatic animals, gut microbes have been recognised to play a role in the development, nutrition, immune response and disease resistance of their hosts [12–15]. In shrimp, bacteria may also have several beneficial roles [14–16]. As with other animals, the gut of shrimp are exposed to the environment and as such represents an important portal of entry for pathogens (bacteria and viruses), which then can establish and develop into an infection. When compared to humans and insects, however, virtually nothing is known about the intestinal immunity and the role of the intestinal flora in crustaceans.

The gastrointestinal (GI) tract of shrimp can be divided into three main parts: a foregut, a midgut and a hindgut. The foregut consists of a stomach which is connected to an oesophagus and to the middle of the hepatopancreas. The midgut includes the anterior midgut caecum, the midgut tube, and the posterior midgut caecum. The hindgut connects the posterior midgut caecum to the anus [17]. The foregut and the hindgut are covered by cuticle which acts as a physical protective barrier. The midgut, however, is not lined with cuticle but instead possesses an epithelial layer that is protected by a semi-permeable, non-permanent and sloughable peritrophic matrix (PM) [18].

Vibrios are ubiquitous marine bacteria that are found in a wide range of aquatic habitats, and are frequently encountered in association with marine organisms. Some species of *Vibrio* such as *V*. *parahaemolyticus* and *V*. *cholera* are among those that are of concern in food safety as these are pathogenic bacteria that can cause gastroenteritis in human. Both species of *Vibrio* can produce chitinolytic enzymes and utilize chitin; they are frequently associated with the exoskeletons of Crustacea [19]. Chitin-binding proteins for the attachment of these *Vibrio* species to chitin have been reported in *V*. *harveyi* [20, 21] and *V*. *parahaemolyticus* (*i*.*e*. chitovibrin) [22]. In *P*. *monodon*, vibriosis is a generic term for an infection caused by any number of *Vibrio* species including *V*. *harveyi*, *V*. *vulnificus* and *V*. *parahaemolyticus*, all of which can result in the mass mortality of stock in hatcheries and grow-out ponds [23, 24]. Among these, *V*. *harveyi* is the most virulent and prevalent pathogen of cultured penaeid shrimp [25–27].

Several studies of the gut bacteria from wild and cultured penaeid shrimp have been documented using either the culture-dependent plate count method [28] or culture-independent methods based on 16S rDNA such as PCR-DGGE (denaturing gradient gel electrophoresis) and clone libraries [29–32]. The results from both types of methods consistently show that *Vibrio* spp. are among the most dominant groups of bacteria in the guts of shrimp, although the dominant genera can vary between different farms and localities [31].

Since 2009, AHPNS (Acute Hepatopancreatic Necrosis Syndrome) or EMS (Early Mortality Syndrome) has caused a huge decline in the production of farmed white shrimp (*Litopenaeus vannamei*), resulting in significant economic losses throughout South East Asia. At the time, the causative agent(s) responsible for mortality were unknown and a matter of controversy. Histopathological examination of moribund shrimp, however, showed that the effects of EMS appear to be limited to the hepatopancreas of the gastrointestinal (GI) tract, and only very recently has the causative agent of AHPNS been shown to be *V*. *parahaemolyticus* [33, 34]. This finding indicates that the main route of infection is the GI tract. Little information, however, currently exists in scientific literature regarding the nature of attachment and the localization of these bacteria within the GI tract of *P*. *monodon*. Likewise, no information exists relating to the interaction between pathogenic bacteria and the inner gut surface. The aim of the current study, therefore, was to observe the interaction of pathogenic *Vibrio* and the inner surface of the digestive tract of *P*. *monodon*, with a specific focus on their *in situ* morphology, aggregation and attachment characteristics under normal conditions and when presented with pathogenic bacterial species via their diet.

## Materials and Methods

### The presence of normal flora in un-infected naive *Penaeus monodon*

The presence and attachment of normal flora in the GI tract of *P*. *monodon* was examined by scanning electron microscopy (SEM). One-month old shrimp were obtained from the Marine Technology Research Center, Faculty of Marine Technology, Burapha University Chanthaburi Campus, Thailand. The shrimp were cultured in a 20 × 20 m plastic-lined pond (10–11 ppt salinity) and fed four times daily with a commercial feed (Starfeeds containing 40% protein). Once the shrimp arrived in the laboratory, apparently healthy individuals were selected for the study. At post-mortem, the entire GI tract including the stomach, midgut and hindgut from several shrimp were prepared for SEM.

### The interaction of pathogenic and non-pathogenic bacteria with the epithelial surface of the GI tract of *Penaeus monodon*

The interaction of both pathogenic and non-pathogenic bacteria with the epithelial surface of the GI tract of *P*. *monodon* was assessed by SEM. For this, one month old shrimp (average 2–3 g body weight) were obtained from a commercial shrimp farm in Pathumthani Province and then transported to Chulalongkorn University where they were maintained in tanks with running, aerated 5ppt water at ambient temperature (28 ± 2°C).

#### Oral route of infection with the delivery of bacteria via an Artemia diet

Shrimp were infected orally by feeding on *Artemia* that were allowed to filter feed on different bacteria before the *Artemia* were presented to the shrimp. Two pathogenic and two non-pathogenic bacterial isolates were selected. *Vibrio harveyi* 1526 [35] and *V*. *parahaemolyticus*, previously isolated from wounded *P*. *monodon*, were chosen as representatives of shrimp pathogens, whilst *Micrococcus luteus* Ml 11 [36] and *Vibrio* B4-24, closely related to *V*. *sagamiensis* based on 16S rDNA and isolated from intestines of broodstock shrimp, served as two non-pathogenic species for assessment. Each bacterial isolate was cultured separately in sterile tryptone soya broth (TSB, Oxoid) supplemented with 2% (W/V) NaCl, except for the *M*. *luteus* which was cultured without a NaCl supplement. The isolates were grown for 18 h (28°C for the *Vibrio* spp. and 30°C for *M*. *luteus*) with constant shaking (250 revolutions/ min). The bacterial cells were subsequently pelleted by centrifugation (3,500 g for 10 min at 4°C), washed twice using sterile 0.9 or 2% NaCl, and then re-suspended in sterile 0.9% or 2% NaCl. The concentration of bacteria within the suspension was adjusted to an absorbance of ca. 1.0 at OD 600 nm (*i*.*e*. approximately 1 × 10^8^ CFUs/ml). Sixty *Artemia* were then allowed to filter feed on each bacterial suspension for 30 min before they were presented to the shrimp. Feeding were monitored for 60 minute to ensure that the shrimp consumed all 60 *Artemia*.

Individual shrimp were placed in 5 L plastic boxes each containing 1.5 L of 5 ppt salinity seawater. Each shrimp were fed once with 60 *Artemia*. Three shrimp from each treatment group were collected at 1.5, 6 and 24 h post infection and then processed for SEM. Additionally, shrimp were collected at 24 h to confirm shrimp infection by quantitative real-time PCR. The bacterial concentrations of the pre-soaked *Artemia* were determined on TCBS agar. One *Artemia* was subsequently found to contain approximately 10^7^ CFU of bacteria, and hence each shrimp received approximately 6 × 10^8^ CFUs of bacteria /shrimp. No sign of molting nor unexpected mortality occurred before the final collection at 24h.

#### Scanning electron microscopy (SEM)

The GI tract from each *P*. *monodon* was dissected out and then fixed in 3% glutaraldehyde in 0.1M phosphate buffer pH 7.4. The samples were stored in the dark overnight at 4°C and then they were rinsed twice with 0.1 M phosphate buffer for 10 min followed by once with distilled water for 10 min. Thereafter they were dehydrated through a graded ethanol series, *i*.*e*. 10 min each in 30, 50, 70, 95% followed by 10 min each in three changes of absolute ethanol. The samples were subsequently critical-point dried using carbon dioxide as the transitional fluid and then mounted on stubs. During mounting, the samples of the foregut, midgut and hindgut were split longitudinally to expose the gut contents and the inner lining of the gut. After sputter coating the samples with gold using a Balzers model SCD 040, the specimens were examined in a JEOL model JSM-5410LV scanning electron microscope.

### Real-time PCR quantifying the concentration of *Vh* and *Vp*

Real-time PCR was used to quantify the concentration of *Vh* and *Vp* in the GI tract of each shrimp after oral delivery of the bacteria via *Artemia*. In triplicate, the stomach and the midgut were collected from control (unchallenged) and challenged shrimp at 24 h post infection. Genomic DNA were extracted using QIAamp DNA blood mini kit (Qiagen) based on the instructions of the manufacturer. The concentration of *Vp* was confirmed by *Vp-*specific primers targeting 2 genes: *gyr B* (gyrase B) and *tlh* (thermolabile hemolysin), and the concentration of *Vh* was confirmed by *Vh*-specific primers targeting the *gyr B* gene. All primer sets that were used in this study are listed in Table 1.

**Table 1:** List of real-time PCR primers to detect the stomach and midgut bacteria.

Target organism	Primer name	Sequence (5′ to 3′)	Product size (bp)	Ta (°C)	Reference
**All bacteria**	Eub338	ACTCCTACGGGAGGCAGCAG	181	55	[37]
	Eub518	ATTACCGCGGCTGCTGG			
***Vibrio harveyi***	A2	TCTAACTATCCACCGCGG	362	64	[38]
	B3	AGCAATGCCATCTTCACGTTC			
***Vibrio parahaemolyticus***	VP-1	CGGCGTGGGTGTTTCGGTAGT	385	58	[39]
	VP-2r	TCCGCTTCGCGCTCATCAATA			
	tlh-f	ACTCAACACAAGAAGAGATCGACAA	207	60	[40]
	tlh-R	GATGAGCGGTTGATGTCCAA			

External standard curves of all genes were constructed using recombinant plasmid of PCR product of each gene inserted into pGEM T easy vector (Promega). Plasmid DNA was used as the template to estimate the copy number of each gene [41]. A 10-fold serial dilution of a known copy number was prepared corresponding to 10^3^−10^8^ copy number/μl for standard curve amplification. All real-time PCR reactions were carried out in a 96 well plate and each sample was amplified in duplicate using a LightCycler 480 II system (Roche). LightCycler 480 SYBR Green I Master (Roche) and 0.2 μM primer concentration were used for real-time PCR amplification. The thermal profile for real-time PCR was 95°C for 10 min followed by 50 cycles of 95°C for 15 s, 55–64°C for 30 s and 72°C for 30 s. The ratio of each gene and 16s rDNA was calculated to express the relative of *Vh* or *Vp* and all bacteria in the stomach and midgut of shrimp. The independent sample t-test (*p*<0.05) was statistically analyzed using SPSS 13.0 for windows. Since there can be multiple copies of 16S rDNA per genome of bacteria (1–13 copies in all bacteria) [42], we assumed for calculation purpose that the average copy number of 16S rDNA was 10.25, based on the average 16S rDNA copy number of *Vp* and *Vh* (11 for *Vp* and 8–11 for *Vh*) [43, 44].

## Results

### General features of shrimp GI tract

**Fig 1:**
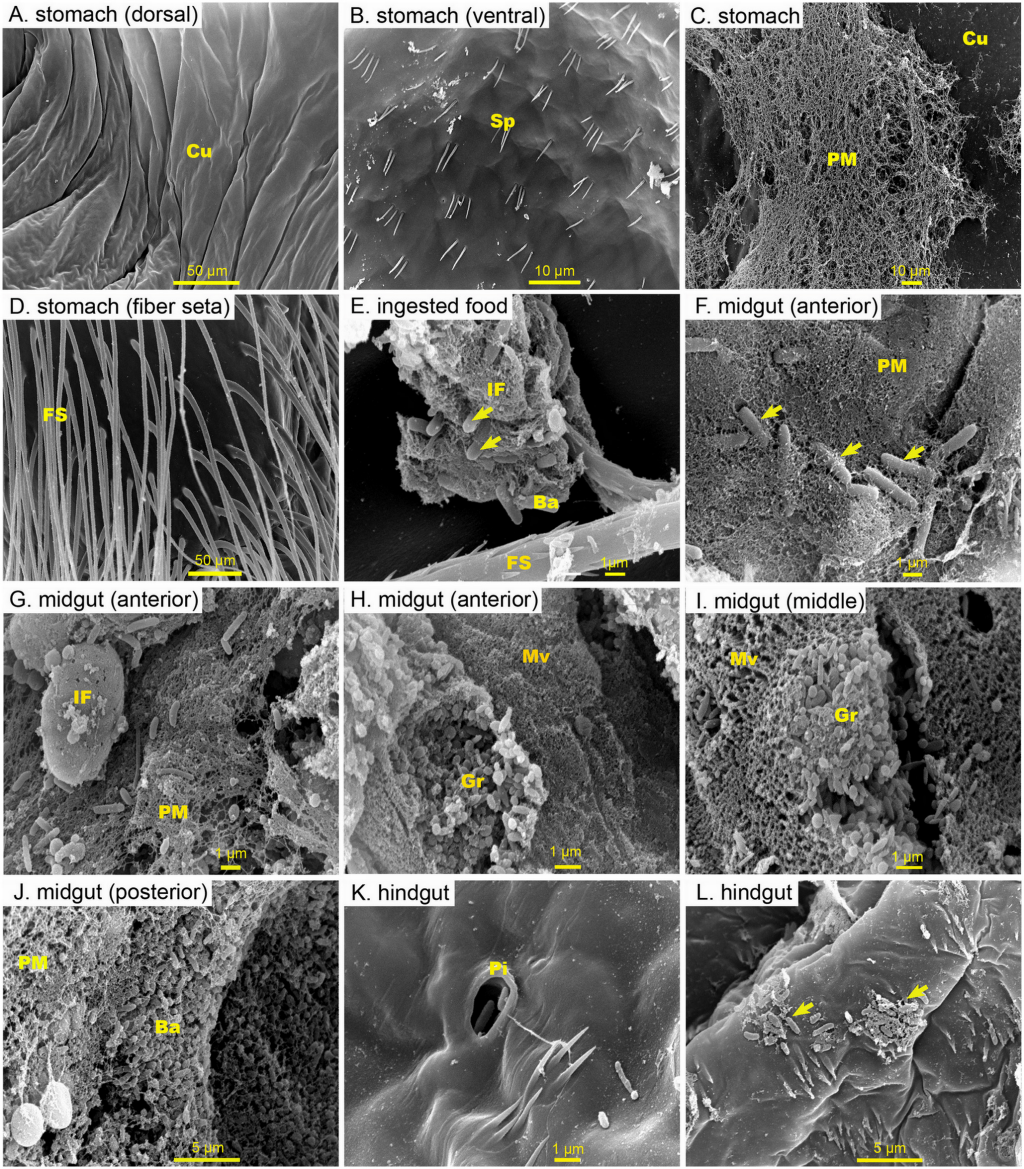
Representative scanning electron microscopy (SEM) micrographs of the inner surface of the digestive of farmed *Penaeus monodon*. Inner surface of (A) the dorsal (B) ventral, (C) peritrophic membrane, and (D) fiber seta of the stomach were devoid of bacteria. (E) bacterial cells (arrowed), however, were seen in association with food inside the stomach. (F, G, H, I, J) Healthy midgut have intact microvilli, and a large number of bacteria were observed attached to the peritrophic membrane and food particle in the midgut. (F, I) Massive granules among the epithelial cells can be seen projecting into the midgut lumen between the microvilli. (K, L) only a few bacteria were seen attached to the cuticle lining of the hindgut. Abbreviation: cuticle (cu), spines (Sp), peritrophic membrane (PM), fiber seta (FS), ingested food (IF), bacteria (Ba), microvilli (Mv), granule (Gr), pit (Pi)

The GI tract of *P*. *monodon* consists of three main segments: a foregut, a midgut and, a hindgut. The midgut is the longest segment of the GI tract running from the posterior end of the pyloric stomach to the hindgut, and then to the anus. It is also connected to the hepatopancreas, the anterior-dorsal digestive caecum and the posterior-dorsal digestive caecum. The foregut and the hindgut originate from stomodeal and protodeal ectoderm, respectively, while the midgut is derived from endoderm. The inner surfaces throughout the foregut and hindgut are lined by cuticle (Fig 1A–1D, 1K and 1L), but the inner surface of the midgut is not (Fig 1F–1J).

### Presence of a normal flora in the GI tract of pond-cultured *Penaeus monodon*

SEM observations (n = 4 shrimp) showed that the inner surface of the stomach is devoid of bacterial cells (Fig 1A–1D), and that in the stomach bacterial cells were only found in association with ingested feed (Fig 1E). Bacterial cells were found singly scattered on the PM of the midgut (Fig 1F and 1G), and large bacterial clusters were seen embedded in the PM within the posterior segment of the GI tract (Fig 1J). No bacteria were observed to attach to the brush border of the midgut lumen or were seen in the ectoperitrophic space (between the PM and the midgut epithelium). A cluster of granules inside the cytoplasm of the epithelial cell were seen protruding through the microvilli into the lumen of the midgut (Fig 1H and 1I). The hindgut was observed to have a thick folded epithelium and a thin immature peritrophic membrane (Fig 1L). The posterior part of the hindgut or the rectum was also lined with cuticle with backward projecting spines (Fig 1K and 1L). A few bacterial cells were seen within the hindgut; these were principally short-rod shaped bacteria attaching to the inner surface or in small pits scattered on the inner surface of the hindgut. (Fig 1K and 1L).

**Fig 2:**
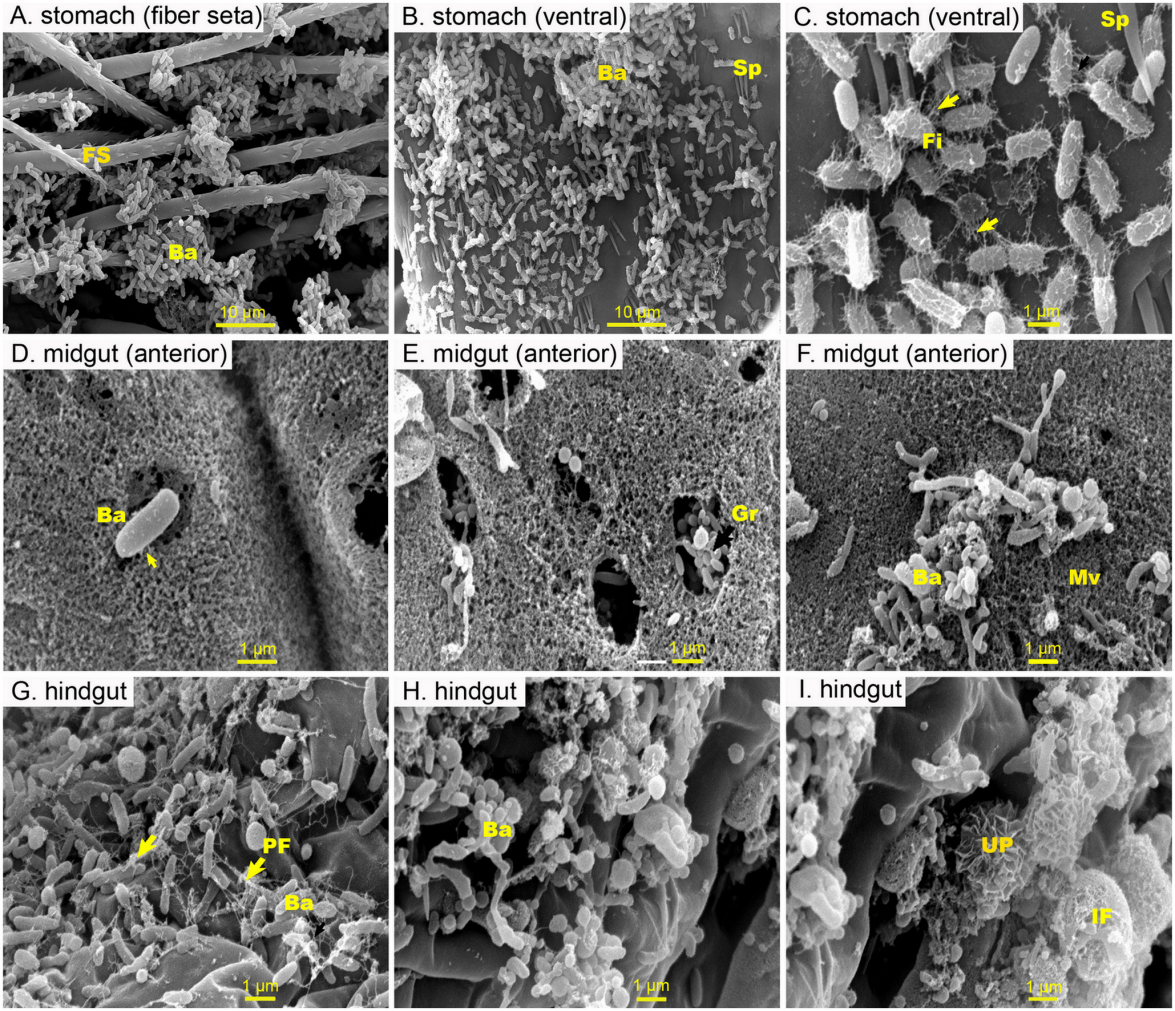
Representative SEM pictures of the inner surface of the digestive tract of a suspected diseased *Penaeus monodon* from a shrimp farm. A cluster of unique rod-shaped bacteria attached to (A) fibre setae or (B) to the lining of the stomach. (C) Higher magnification image of the attached bacteria in the stomach exhibiting peritrichous pili-like structures or fimbria., where (D) a few fibres linked to the peritrophic matrix (PM) can be seen (arrowhead). (E) Many holes were created in the PM and a few granules were seen inside the holes. (F) A group of irregular-shaped bacteria were found attached to the PM. (G) A cluster of short-rod shaped bacteria with polar flagella, (h) irregular-shaped, and (I) unidentified particles were seen attached to the hindgut wall. Abbreviation: fiber seta (FS), bacteria (Ba), spines (Sp), fimbria (Fi) granule (Gr), microvilli (Mv), polar flagella (PF), unknown particles (UP), ingested food (IF)

Of particular interest is one shrimp specimen that had patches of unique rod-shaped bacterial population firmly attached to the fibre setae (Fig 2A) or to the stomach lining (Fig 2B). The attached bacteria exhibited peritrichous pili-like structures or fimbria (Fig 2C), and a few fibres were seen linked to the PM (Fig 2D). In addition, these bacteria had the ability to degrade the PM, as evident by the presence of numerous holes in the PM and the exposure of cytoplasmic granules of the epithelial cells under the PM (Fig 2E). In addition to these, another group of irregular-shaped bacteria were found attached to the PM (Fig 2F). In the hindgut of the same shrimp, a cluster of short-rod shaped bacteria with polar flagella and irregular-shaped and non-identifiable particles were observed adhering to the wall of the hindgut (Fig 2G–2I).

### Colonization of pathogenic *Vibrio* and the intestinal pathology they induce

**Fig 3:**
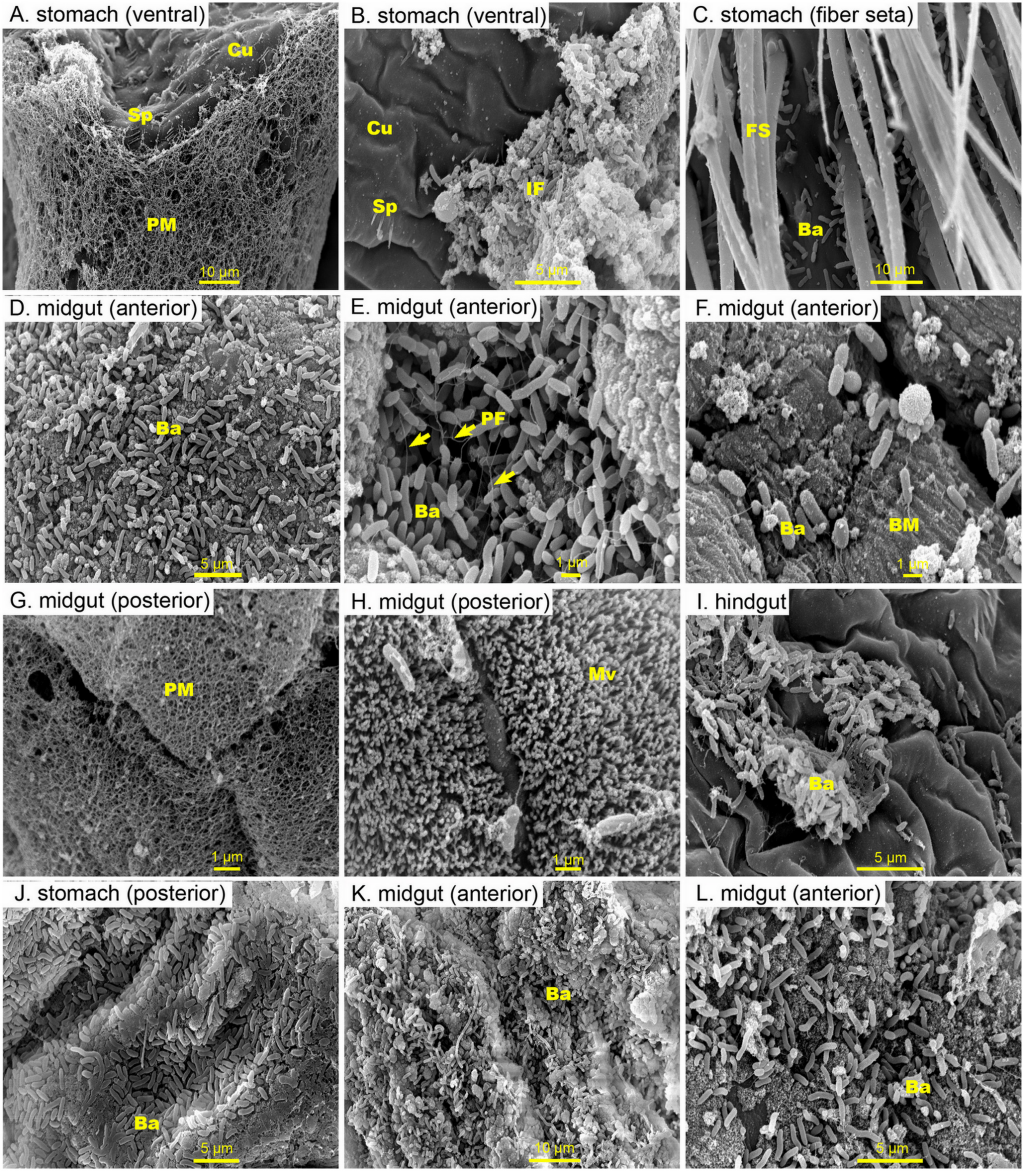
Representative SEM images of the inner surface of *P*. *monodon* infected with *Vibrio harveyi*. (A) At 1.5 h post-infection (PI), no bacteria adhering to the surface of the stomach linings were seen, but (B) numerous bacterial cells mixed with ingested food attaching the stomach surface were seen. (C) Numerous rod-shaped bacteria firmly attached to the stomach lining. (D) At 6 h PI, colonizing bacteria cover the epithelium of the anterior midgut. (E, yellow arrow) A higher magnification of the bacteria seen at 6 h show that they possess polar flagella that are linked with each other, and (F) heavy destruction of the epithelial layers by bacteria exposed of the basement membrane underneath. (G) The posterior portion of the midgut showing intact tissue with a thick peritrophic matrix or (H) with a few bacterial cells attached to the microvilli. (I) Scattered clusters of rod-shaped bacteria adhering to the lining of the hindgut. (J-L) At 24 h PI, the numbers of bacteria within the stomach of infected shrimp increased dramatically. Densely packed-bacteria were found covering the epithelium of the anterior midgut. Abbreviation: cuticle (Cu), spines (Sp), peritrophic membrance (PM), ingested food (IF), fiber seta (FS), polar flagella (PF), bacteria (Ba), basement membrane (BM), microvilli (Mv)

The progression of *Vh*- or *Vp-*induced pathological changes in the luminal surface tissues of the stomach, midgut and hindgut in infected shrimp at 1.5, 6 and 24 h post-infection (PI) were visualised by SEM and compared. At 1.5 h PI with *Vh*, no bacteria were seen adhering to the surface of the stomach (Fig 3A), but numerous bacterial cells mixed with ingested food were found loosely attached to the lining of the stomach lumen (Fig 3B). At 6 h PI, numerous rod-shaped bacteria of a single morphotype were found firmly attached to the stomach surface in places (Fig 3C) and to the upper and middle regions of the midgut (Fig 3D and 3E). The epithelial layers with colonizing bacteria exhibited signs of destruction in both the stomach and the upper midgut, whereas the areas further down the midgut to the hindgut without bacterial colonization were still intact (Fig 3G). Bacterial replication continued between 6 and 24 h PI, indicating that the bacterial populations were growing *in situ*. At 24 h PI, bacterial numbers dramatically increased within the stomach (Fig 3J) and persisted in the posterior part of the midgut (Fig 3K). Extensive, severe destruction of the epithelium in the upper midgut at 24 h PI was observed under the colonized bacterial mat as indicated by the disappearance of the epithelial layer and the exposure of the underlying basement membrane (Fig 3F–3I). At this time point, however, most of the epithelium of the posterior midgut and hindgut remained intact (Fig 3H and 3I). The posterior part of the midgut was free of PM with some bacterial cells seen attached to the microvilli (Fig 3H), whilst the area between the hindgut and the midgut was covered by a very thick PM (Fig 3G). Scattered clusters of rod-shaped bacteria were also seen within the hindgut (Fig 3I), but it appears as if they were not detrimental to the host.

**Fig 4:**
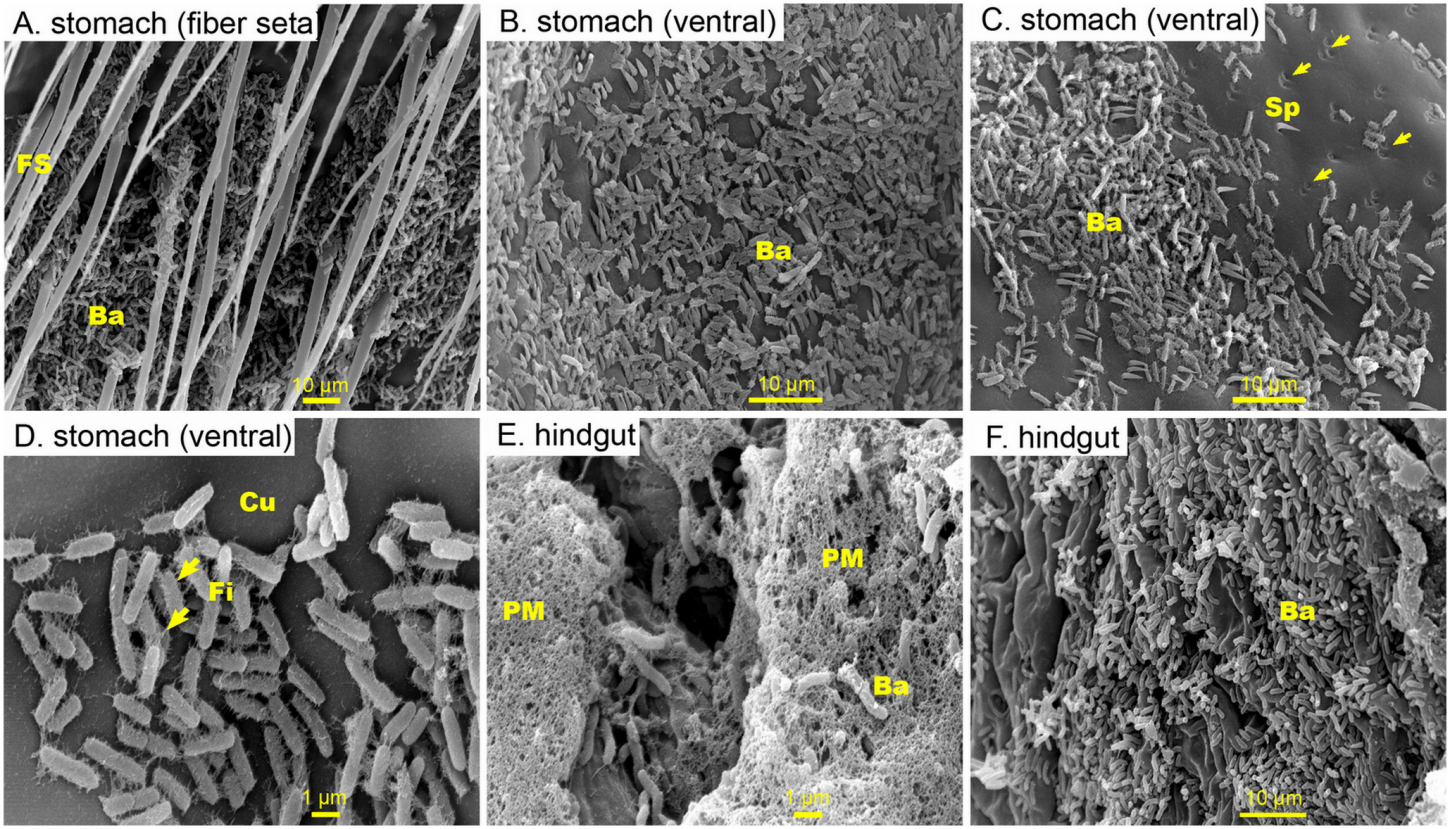
Representative SEM images of the inner surface of the digestive tract of *P*. *monodon* infected with *Vibrio parahaemolyticus*. (A) At 24 post-infection, numerous straight-shaped bacteria adhering to the fibre seta, to (B) short spines and (C) to the inner surface of the stomach. Some of the spines were broken and had detached from the stomach lining (arrowheads). (D-F) Attached bacteria producing peritrichous pili-like structures. Abbreviation: fiber seta (FS), spines (Sp), cuticle (Cu), fimbria (Fi), peritrophic membrane (PM)

In shrimp fed *Vp*, numerous straight-shaped bacteria (~1.8–2.2 μm in length) were seen attached to the fibre seta, to the short spines and to the inner surface of the stomach at 24 h PI (Fig 4A–4C). No severely damaged tissues were seen except some broken and detached spines from the stomach lining (Fig 4C). Attached bacteria within the stomach also produced peritrichous pili-like structures (Fig 4D). The posterior part of the midgut and the hindgut, however, were extensively colonized by rod-shaped bacteria which differed morphologically from the attached bacteria within the stomach as no peritrichous pili-like fibres were observed (Fig 4E and 4F).

**Fig 5:**
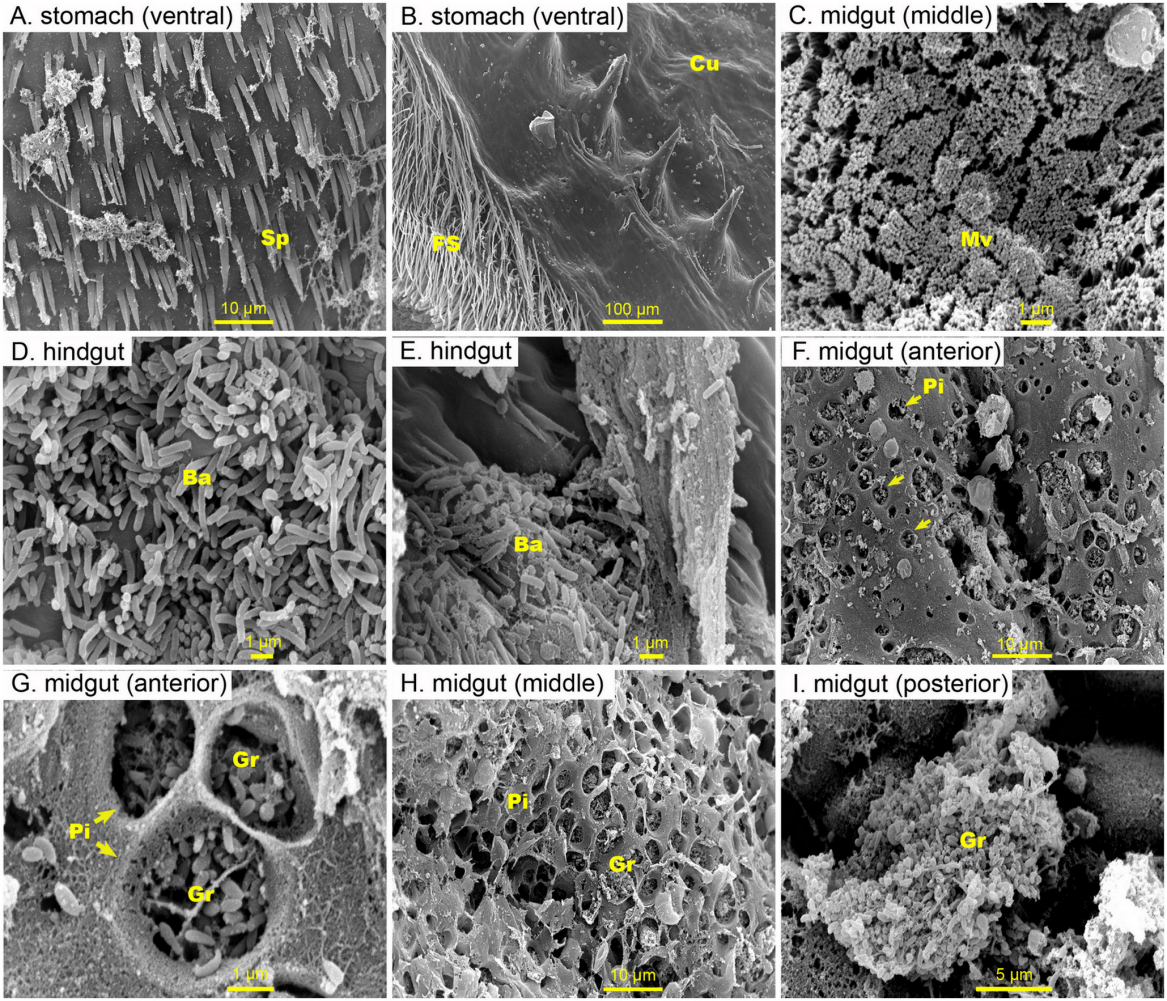
Representative SEM images of the inner surface of the digestive tract from *P*. *monodon* receiving non-pathogenic bacteria (*Micrococcus luteus* and non-pathogenic *Vibrio* B4-24). No observed attachment of bacteria to the stomach (A—*M*. *luteus*, B- *Vibrio* B4-24) or to the midgut (C- *Vibrio* B4-24). The bacteria found in the hindgut were variable in number where most of the hindgut tissues were intact (D—*M*. *luteus*, E- *Vibrio* B4-24). A high number of pits were found across the surface of the midgut (G, H- *Vibrio* B4-24), where a large number of cocci- and spindle-shaped granules which resided in the epithelial cells were seen (I- *Vibrio* B4-24). Abbreviation: spines (Sp), fiber seta (FS), cuticle (Cu), microvilli (Mv), bacteria (Ba), pit (Pi), granule (Gr)

In the GI tract of *P*. *monodon* fed non-pathogenic bacteria, *i*.*e*. *M*. *luteus* and *Vibrio* B4-24, the bacteria were seen only on the hindgut lining (Fig 5D and 5E) and not on the stomach surface (Fig 5A and 5D) nor on the epithelium of the midgut (Fig 5E). In nearly all the shrimp that were examined, a high number of pits, measuring 2–5.5 μm in diameter, were found across the surface of the midgut (Fig 5F and 5G). Inside each pit, a massive number of cocci- and spindle-shaped granules, which normally reside in the cytoplasm of the epithelial cells, were observed in the anterior midgut (Fig 5H). Similar granules of larger size were observed further along the digestive tract in the posterior midgut (Fig 5I). Notably, the pit number varied among individual shrimp but was not correlated with the degree of infection. Among all examined shrimp, the hindgut bacteria were mostly varied in the number of bacteria observed, while the morphotypes were similar. Most of the hindgut tissues were intact (Figs 1H and 1I, 2G–2L, 3I, 4F and 5D–5E).

### The concentration of *Vh* and *Vp* in the GI tract of challenged shrimp

**Fig 6:**
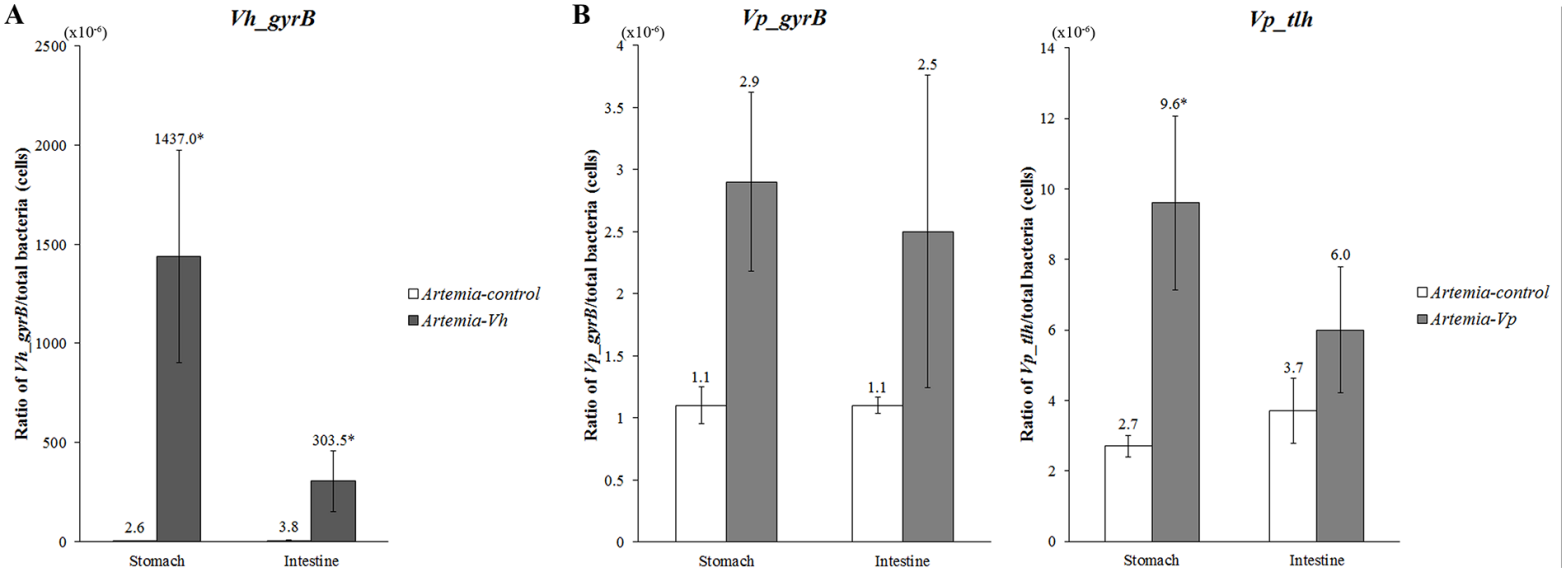
Real-time PCR presented of the ratio of the *gyrB* gene of *Vibrio harveyi* (A) and 16S rDNA bacteria, and the ratio of the *gyrB* and *tlh* gene of *Vibrio parahaemolyticus* (B) and 16S rDNA bacteria in the GI tract of *P*. *monodon*. Error bars were expressed as ±SEM. Asterisk indicates significant differences between the control and challenged group (*P*<0.05).

Real-time PCR was used to confirm and quantify the presence of *Vh* and *Vp* specific genes (relative to 16S rRNA gene) in the stomach and the midgut at 24 h post challenge (Fig 6A). The ratio of *Vh_gyrB* to total bacteria in both stomach and midgut of the challenged shrimp (1437 x 10^−6^ and 303.5 x 10^−6^, respectively) were significantly higher than that of the control group (*P*<0.05)(2.6 x 10^−6^ and 3.8 x 10^−6^, respectively). The ratio of *Vp_gyrB* to total bacteria in the stomach and the midgut of challenged shrimp (2.9 x 10^−6^ and 2.5 x 10^−6^, respectively) was higher than that of the control shrimp (1.1 x 10^−6^ and 1.1 x 10^−6^, respectively). Similarly, the ratio of *Vp_tlh* to total bacteria significantly increased in the stomach of challenged shrimp (9.6 x 10^−6^) compared to that of the control unchallenged shrimp (*P*<0.05) (2.7 x 10^−6^). The midgut of challenged shrimp also showed higher ratio of *Vp_tlh* to total bacteria (6.0 x 10^−6^) than that of the unchallenged ones (3.7 x 10^−6^), but the difference was not significant.

## Discussion

The present work provides a description of the interaction between two pathogens, *V*. *harveyi* (*Vh*) or *V*. *parahaemolyticus* (*Vp*), and the inner surface of the GI tract of farmed *P*. *monodon* following infections via the oral route, with comments on their appearance, their attachment and their colonization sites based on SEM observations. In healthy shrimp, few bacteria were found attached to the inner surface of the stomach or to the midgut, but bacteria could be observed in the posterior midgut and hindgut. Some bacteria were also seen in association with ingested feed. To show that shrimp pathogens like *Vh* and *Vp* are capable of colonizing the lining of the digestive tract, a simple oral infection model of pathogenic bacteria-induced gut pathology was used in the current study. This approach to bacterial challenge better resembled an actual infection via the digestive tract in aquaculture settings, and helped gain an understanding of key features of the pathogenesis in shrimp elicited by *Vh* and *Vp* in the digestive tract.

In *P*. *monodon* infected with two pathogenic *Vibrio* species, attachment and colonization of bacteria on the stomach and hindgut lining were observed. The higher number of copies of *Vp* and *Vh* specific genes in the stomach and midgut of challenged shrimp compared to that of the control shrimp (Fig 6) suggested that *Vp* and *Vh* from the challenge became established in the GI tract of shrimp. *Vibrio spp*. are a natural part of the GI microbiota of shrimp, and some species can cause opportunistic infection during adverse culture conditions. In other studies, both *Vh* and *Vp* had been found in apparently healthy farmed-raised shrimp showing no symptoms [45–50]. Low but detectable level of *Vh* and *Vp* was found in the GI tract of control (unchallenged) shrimp in the present experiment, but their concentration was lower than that of the challenged shrimp. In addition to the higher concentration of *Vh* and *Vp*, the higher virulence of the introduced *Vh* and *Vp* strains compared to that of the endogenous ones may explain the observed attachment and colonization in the challenged group. Since the concentration of *Vh* and *Vp* in this experiment was determined by specific PCR of only one (*Vh)* or two (*Vp)* genes, other virulent factors associated with either introduced or endogenous *Vh* and *Vp* were not examined. The difference in virulence with-in a species of clinical *Vp* had been reported in the literature. Using multilocus sequence analysis, Theethakaew et al [51] reported a distinct cluster of human pathogenic *Vp* isolates among other environmental *Vp* isolates in the environment and suggested a high degree of genetic diversity within the species. Other *in situ* techniques such as Fluorescent *in situ* Hybridization (FISH) could provide the most definitive evidence associated *Vh* or *Vp* at infection sites. At present, FISH analysis of shrimp's stomach and intestinal tissues remains difficult due to auto-fluorescence [52, 53].

A few bacterial cells were found to adhere to the peritrophic membrane (PM) or to the gut contents throughout the midgut, except for the posterior end of the midgut where it is connected to the hindgut (Fig 2A, 2B, 2E, 2E, 2H and 2I). No bacterial cells were observed between the PM and the epithelium of the midgut or the ectoperitrophic space. The absence of bacterial flora in the ectopertrophic space of the midgut in *P*. *monodon* in the present study is consistent with observations reported in other marine crustaceans. Martin *et al*. [18] demonstrated that no bacteria were associated with the brush border or were in the ectoperitrophic space of the midgut of ridgeback prawn *Sicyonia ingentis*. Likewise no evidence of bacterial colonization in the midgut of mud shrimps *Calocaris macandreae* was reported [54]. Bacteria capable of attachment to the midgut area could potentially be long-term residents in the midgut due to the fact that this area is not shed during molting. Similar observation was made in an SEM and TEM study of the digestive tract of the hydrothermal vent amphipod *Ventiella sulfuris* [55]. The study speculated that long rod-shaped bacteria found between the microvillous epithelial cells could be considered as long-term residents within the bacterial community of the midgut due to its locality in the midgut and the healthy appearance of epithelia in contact with the bacteria [55].

In insects and crustaceans, the lining of the GI lumen consists of a thin but tough peritrophic membrane which is quite unlike the thick mucosa seen in mammalian guts. The PM is a semi-permeable, non-cellular structure, which surrounds the food bolus and is composed of chitin, glycoproteins and mucins, providing a chemical and physical barrier against infection by ingested pathogens [18, 56, 57]. The PM of the *P*. *monodon* specimens used in the present study are similar to those described in other penaeid shrimp, *e*.*g*. *S*. *ingentis*, which has very small pores that will allow only inert particles less than 20 nm to pass through the PM [18]. Although the PM can provide resistance to pathogenic bacteria, *e*.*g*. as in *Daphnia magna* [58]; can trap enteropathogenic *Aeromonas caviae* in houseflies *Musca domestica* [59]; and, can limit infection by baculovirus in a moth *Trichoplausia ni* larvae [60], some bacteria can secrete enzymes to degrade the PM resulting in large holes in the membrane allowing bacteria to subsequently colonize the epithelial layer [61]. Moreover, some pathogens such as *V*. *parahaemolyticus* do not need to penetrate the PM to cause damage because they can produce toxins that are able to pass through the PM of *S*. *ingentis* [62]. The presence of a PM is one reason why there are no bacteria associated with the brush border of the epithelium; another reason can be that the mucosal immunity within the shrimp’s gut may directly and tightly control the number of microbes [63].

Although all *P*. *monodon* specimens that were used in this study were collected from a commercial farm and were apparently healthy, one specimen was found to have an abundance of a single morphotype rod-shaped bacterium with fimbria-like fibres extensively adhering to the cuticular lining of the stomach. The inner surfaces of the stomachs from the other four specimens of *P*. *monodon* that were collected at the same time were devoid of microbes. As it is in other animals, an important property of a pathogenic bacterium in shrimp is its ability to gain an appropriate attachment as this is the initial step in the infection process. It is possible that this particular specimen of *P*. *monodon* may have already been infected by pathogenic bacteria, since it is unusual that ingested microbes can colonize and establish within a harsh environment as the stomach.

All *P*. *monodon* used in this study were seen to have bacteria attaching to the surface of the hindgut or to the posterior part of the midgut wall where it connects with the start of the hindgut. These findings support those of Harris [12] who reported that the presence of bacteria in the hindguts of Crustacea are widespread, occurring throughout taxa belonging to marine Thalassinidae and Brachyura (9 genera, 16 species). There was, however, a high degree of variability between specimens in both the types and the total numbers of bacteria in the hindguts, indicating that this bacterial population may be regulated by its host. It has been suggested that molting may have a direct influence on the bacterial communities within the hindgut [64, 65]. For each molting cycle of the exoskeleton, the chitinous hindgut lining is displaced and replaced with a new lining [66]. While there is no report in the literature on the effect of molting on the hindgut microbiota of shrimp, the newly molted hindgut surface was shown to be devoid of microbes in a study on desert millipedes *Orthoporus ornatus* (Girard) [67]. It is not currently known how microbes recolonize in the hindgut after molting. From our observations in penaeid shrimp, however, it can be hypothesized that the bacteria attached to the posterior part of the midgut, *i*.*e*. immediately adjacent to the hindgut, can function as a bacterial inoculum. The presence of bacteria in the posterior midgut in our study supports this hypothesis.

The hindgut environment with its chitinous lining has been shown to be a suitable place for the colonization of bacteria. Not only there are preference for hindgut attachment in some species of bacteria, but there also appears to be some selective pressure from the host on the types of bacteria present in the hindgut. For example, *V*. *cholerae* has been reported to preferentially attach to the hindgut of the blue crab *Callinectes sapidus*, but not to the midgut [68]. Selective pressure from Bay ghost shrimp *Neotrypaea californiensis* was shown to limit the number of bacterial taxa present in its hindgut [69]. It is likely that hindgut bacterial community in the gut of *P*. *monodon* is specific this host and that the majority of bacteria that are attached are *Vibrio* spp. The dominance of *Vibrio* spp. in the GI tract of *P*. *monodon* has been shown in several studies looking at the bacterial communities in both wild and cultured populations of shrimp [28–31]. Similarly, *Vibrio* spp. were shown to be dominant in the hindgut of *L*. *vannamei* [29].

Adhesion of pathogenic bacteria to the inner surface of the GI tract is crucial to the subsequent establishment of an infection, however, the manner to which *Vh* and *Vp* attach the epithelial surface of their host is very different from each other. From the results, it is most likely that *Vh* binds to the surface of the stomach and to the epithelial layer of the midgut, while *Vp* only colonizes the surface of the stomach. In addition, *Vp* colonizes as a monolayer of cells, while *Vh* colonizes as a multilayered cluster of bacteria. Similar preferences for the site of adhesion have been seen in host-bacteria interactions. The ability of *V*. *cholerae* to colonize the hindgut surface of the blue crab *Callinectes sapidus*, but not the midgut has been shown, led authors of the study to suggest that chitin was required in the attachment of *V*. *cholerae* to invertebrate and zooplankton surfaces [68]. In crustaceans and other arthropods, the preferred area for bacterial establishment is the hindgut, which is covered by a chitinous cuticle that provides anchoring surfaces for bacteria and favours symbiotic interactions [69, 70]. It has been suggested that in natural marine systems, most bacteria attaching to chitinaceous particles are *Vibrios* [71]. Living crustacean surfaces which possess chitinous components are noted for supporting bacterial attachment and growth [72].

Production of flagella or pili by intestinal bacteria can have an important role in colonization and infection. Induction of peritrichous flagella is associated with conversion of *Vp* from small (av. 2–3 μm long), polarly flagellated swimmer cells to swarmer cells, which are elongated (av. 5–20 um long) as well as peritrichously flagellated [73, 74]. Interestingly, we observed lateral cell appendages were produced by the attached *Vp* in the present experiment, suggesting that these cells have switched from the swimmer cell to the swarmer cell state. Many pathogenic bacteria such as pathogenic *E*. *coli* exhibit pili or fimbriae that facilitate their initial attachment to epithelial cells and subsequent successful colonization of their host [75, 76]. Pili are virulence factors that mediate interbacterial aggregation and biofilm formation, or mediate the specific recognition of host-cell receptors [77]. It is clear that pili play similar biological roles for commensal bacteria because they also have to colonize specific niches and overcome the host’s natural clearing mechanisms. Although *Vp* is a leading cause of life-threatening gastroenteritis in human, it is most likely that *Vp* exhibits lower virulence and has a slower proliferation than *Vh* in *P*. *monodon*, as indicated by the lack of infection in the midgut at 24 h PI. This might be because the microenvironment in the gut of *P*. *monodon* is not optimal or suitable for *Vp*.

Our observations confirm that both *Vp* and *Vh* in the present study are shrimp pathogens and their attachment properties, that are a prerequisite for the pathogenesis, are similar to those of other pathogens. The typical infectious cycle of these pathogens in *P*. *monodon* includes: 1) entry of the pathogen through the oral route; 2) bind to chitin on the stomach lining or to the midgut peritrophic membrane, multiply and cause damage to the host tissues; and, 3) exit from the host. Each step would involve adhesion, chemotaxis, production of various lytic enzymes such as haemolysis, secretion systems of the type II or type III secretion system, biofilm formation, and production of a quorum sensing system [78]. Examination of all the *P*. *monodon* hindguts in the current study, including the infected specimen, found numerous bacteria attached randomly across the hindgut luminal surface, both singly and in large, biofilm like microcolonies, some of which contained mats of bacterial cells. Since the morphotype of the bacteria attached in the hindgut differed from *V*. *harveyi* and the *V*. *parahaemolyticus* morphotype, and that the posterior region adjacent to the upper part of the hindgut was not infected by either of these bacteria, the attached bacteria in the hindgut could be considered to be resident and not pathogenic. All the evidence indicates that these bacteria are not pathogenic and that their presence is not detrimental to the host tissues. If these hindgut bacteria are part of the normal bacterialcommunity in *P*. *monodon*, then their putative roles or beneficial effects to their host needs to be elucidated.

## Conclusion

To understand the attachment and localization of bacteria in guts of *P*. *monodon* during infection, the inner surfaces of the gastrointestinal tract of healthy and infected shrimp, *i*.*e*. *V*. *harveyi* and *V*. *parahaemolyticus* via an *Artemia* diet, were examined and compared using SEM. Both pathogens can pass through the oral route and can resist and survive in the stomach. Upon reaching the posterior stomach and the upper and middle portions of the midgut, both pathogens can establish, proliferate, and cause tissue damage, particularly to the epithelial layers. No tissue damage, however, was seen in the posterior midgut or in the hindgut. Infection via the oral route represents a more natural way of infection rather than by the injection of bacteria into shrimp. The findings from this study will enable further research to decipher the bacterial pathogen-host interactions that contribute to disease, leading to new methods to prevent or combat infection, for instance, through inhibition of bacterial attachment, quorum sensing and / or biofilm formation.
